# MAPKs and Signal Transduction in the Control of Gastrointestinal Epithelial Cell Proliferation and Differentiation

**DOI:** 10.3390/ijms140510143

**Published:** 2013-05-13

**Authors:** Luciana H. Osaki, Patrícia Gama

**Affiliations:** Department of Cell and Developmental Biology, Institute of Biomedical Sciences, University of São Paulo, SP 05508-000, Brazil; E-Mail: luciana.osaki@usp.br

**Keywords:** MAPK, cell cycle, differentiation

## Abstract

Mitogen-activated protein kinase (MAPK) pathways are activated by several stimuli and transduce the signal inside cells, generating diverse responses including cell proliferation, differentiation, migration and apoptosis. Each MAPK cascade comprises a series of molecules, and regulation takes place at different levels. They communicate with each other and with additional pathways, creating a signaling network that is important for cell fate determination. In this review, we focus on ERK, JNK, p38 and ERK5, the major MAPKs, and their interactions with PI3K-Akt, TGFβ/Smad and Wnt/β-catenin pathways. More importantly, we describe how MAPKs regulate cell proliferation and differentiation in the rapidly renewing epithelia that lines the gastrointestinal tract and, finally, we highlight the recent findings on nutritional aspects that affect MAPK transduction cascades.

## 1. Introduction

During their life, cells are exposed to several external and internal agents and their combination determines cell fate. Mitogen-activated protein kinases (MAPKs) are important signaling molecules that influence a broad range of cellular processes such as proliferation, differentiation, migration and apoptosis. Since their discovery in the 1980s, MAPKs have been extensively studied (for a historic description, see Avruch [1]) and several reports show that this signaling pathway is highly conserved amongst eukaryotes. In addition, besides its participation in many physiological processes, MAPKs also play important roles in pathological conditions, which include cancer, cardiac hypertrophy and diabetes [2,3].

MAPK pathway is composed of multiple molecules that also transduce signals in other cascades, creating a crosstalk and a wide intracellular network that involves other pathways such as PI3-kinase (PI3K)-Akt, Src, Smad and Wnt-β catenin. However, in addition to these, the variety of stimuli and the specific expression of proteins in the different cell types increase the complexity of MAPK system [4]. In this review, we focused on the role of MAPKs and their major partners in the signaling networks that lead to epithelial cell proliferation and differentiation, and we summarized recent findings on nutritional aspects that can influence MAPK pathway.

## 2. MAPK Signaling Pathway

Activation of MAPKs is part of a signaling cascade that depends on the phosphorylation of molecules up- and downstream within the cell. The source of the signal for MAPK activation can be diverse and includes growth factors, hormones, cytokines and environmental stress, which trigger the phosphorylation of MAP kinase kinase kinase (also referred to as MAPKKK, MEKK or MAP3K) through members of Ras and Rho families of small GTPases. Activated MAPKKK phosphorylates MAP kinase kinase (MAPKK, MEK) which, in turn, phosphorylates MAPK at threonine and tyrosine residues within a conserved Thr-X-Tyr motif in the activation loop [5,6].

Different proteins are part of MAPK group and the most studied are extracellular-regulated kinases 1 and 2 (ERK1 and ERK2), c-Jun NH2-terminal kinases (JNKs), p38 and ERK5. Their ability to specifically recognize their substrates resides in two different properties: proline-directedness and the presence of specific MAPK docking sites [7,8]. Accordingly, MAPKs only phosphorylate serine/threonine residues that are immediately followed by proline, but this structural condition is not enough for the specificity of different MAPKs, and so, substrates also interfere, as they have particular docking sites to which MAPKs are strongly and specifically associated [7,8]. The maintenance of signaling efficiency still depends on scaffold proteins that selectively bind to the multiple components, arranging them into organized modules at special cellular sites [9,10]. Amongst the numerous scaffold proteins involved in MAPK signaling, we should mention: kinase suppressor of Ras 1 (KSR1), MEK partner 1 (MP1), β-arrestin and JNK-interacting partner (JIP1) [9].

MAPK activity is also regulated by a group of phosphatases named MAPK phosphatases (MKPs) that dephosphorylate MAPKs, leading to their inactivation [11]. For example, the dual specificity phosphatase-6 (DUSP-6, also known as MKP-3) is a cytoplasmic MKP that has ERK1/2 as preferential target. Both MKPs and the scaffold proteins mentioned above are important to establish the magnitude and duration of signals, which also determine the output of MAPK pathway stimulation [10,12,13]. The different fates of PC12 pheocromocytoma cells in response to epidermal growth factor (EGF) and nerve growth factor (NGF) are classical examples of temporal control in ERK1/2 signaling, as EGF-induced transient activation of ERK stimulates PC12 cell proliferation, whereas NGF-sustained stimulus leads to cell differentiation [13].

**Figure 1:**
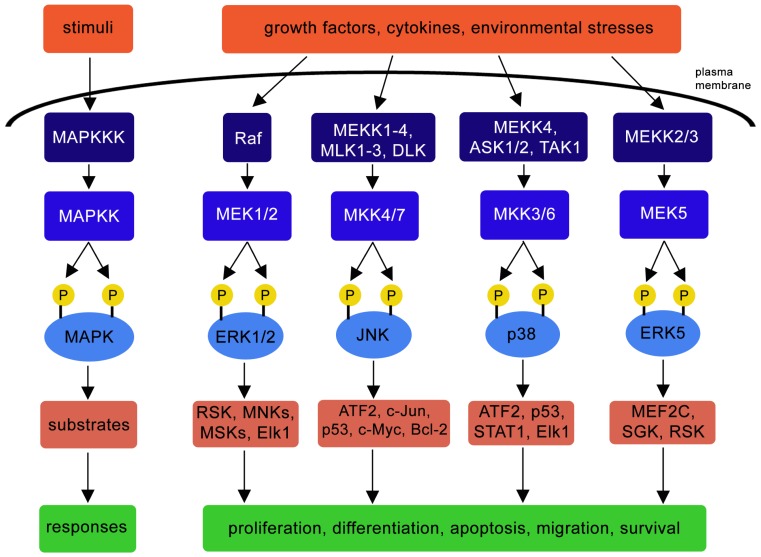
MAPK family, main stimuli, members, targets and effects.

In terms of mechanisms, once activated in the cytosol, MAPK dissociates from anchoring proteins and is rapidly translocated into the nucleus to regulate transcription through the phosphorylation of proteins involved in this cellular event [14] (Figure 1 summarizes the main pathways and the cellular responses). In this way, JNKs, ERKs and p38 control the activity of the transcription factor activator protein-1 (AP-1), and in the stomach of aging rats, the transforming growth factor α (TGFα) induces AP-1 activation in ERK-dependent manner without affecting the other MAPKs [15]. Comparatively, in human proximal tubular HK-2 cells, high glucose concentrations induce p38 activation, which in turn regulate AP-1 response [16].

### 2.1. ERK Pathway

The Ras/Raf/MEK/ERK system is definitely the best characterized MAPK pathway. The canonical ERK cascade starts with the binding of ligands (e.g., EGF and platelet-derived growth factor—PDGF) to transmembrane receptor tyrosine kinases (RTKs). After phosphorylation, RTKs recruit Grb2, a protein that contains Src homology 2 (SH2) domains, which are cytosolic and bound to Son of Sevenless (SOS). SOS is a guanine-nucleotide-exchange factor (GEF) for Ras, a GTPase that exists in humans in three forms: H-Ras, K-Ras and N-Ras. Therefore, SOS is relocated to the plasma membrane, leading to the activation of Ras, which recruits members of the family of MAPKKKs Raf to the membrane, where they are activated. After that, they phosphorylate the MAPKKs (MEK1 and MEK2), that activate ERK1 and ERK2 in the cytosol, which are then translocated into the nucleus, where they can phosphorylate numerous substrates [17]. The output of ERK signal depends on MAPK targets, which are controlled along the whole pathway by many molecules, including the MKPs and scaffold proteins [18].

### 2.2. JNK Pathway

The JNK family of MAPKs has three members in mammals: JNK1, JNK2 and JNK3. This MAPK signaling cascade can be started by many factors, especially environmental stress, genotoxins and cytokines. Both JNK and p38 pathways are also referred to as “stress-activated signaling pathways” (SAPKs). JNKs are phosphorylated by MKK4 and MKK7, which in turn can be activated by several MAPKKKs such as MEKK1-4, MLK1-3 and DLK [5]. Amongst the numerous substrates of JNK are c-Jun, p53, c-Myc, Bcl-2 and Bad [19]. The variability of targets with different functions contributes to the multiple outputs of JNK cascade, *i.e.*, cell death, proliferation, differentiation.

### 2.3. p38 Pathway

There are four members in the p38 MAPK family: p38α (also known as MAPK14), p38β (MAPK11), p38γ (MAPK12) and p38δ (MAPK13). Environmental stress and cytokines are some of the stimuli of p38 pathway, which can be initiated at the MAPKKK level with the phosphorylation of kinases including MEKK4, ASK1, ASK2 and TAK1. The following step is the activation of MKK3 and MKK6 (MAPKKs) that phosphorylate p38, which targets substrates both in the cytoplasm and the nucleus. As a result, the system is connected to diverse cellular processes such as protein degradation, cytoskeleton dynamics, apoptosis and migration [20,21]. In the cytoplasm, p38 can phosphorylate other kinases such as MNK1/2, while in the nucleus, it activates transcription factors as ATF2, p53 e STAT1 [20,21].

### 2.4. ERK5 Pathway

ERK5 was recently described as a member of the MAPK family [22,23], and its pathway can be triggered by stimuli as stress, growth factor and cytokines, which are equally important for the cascades discussed above. Following response initiation, MEKK2 and MEKK3 are activated to phosphorylate MEK5, which directly phosphorylates ERK5, and this MAPK acts on different substrates such as myocyte enhancer factor 2C (MEF2) transcription factor, that culminates in increased expression of c-Jun [24,25].

## 3. Crosstalk with Other Signaling Pathways

MAPK cascades usually do not operate individually, but communicate with each other or with different signaling pathways, generating a wide network. This combination of elements allied to cellular conditions contribute to the determination of MAPK responses upon activation, and as a range of crosstalk possibilities have been identified, we will focus on the interactions among MAPKs and their connections with PI3K-Akt, Smads and Wnt/β-catenin pathways.

The crosstalk among ERK, JNK and p38 takes place at different levels and cellular context [26,27]. p38 downregulates ERK signaling directly or through activity of protein phosphatase 2A (PP2A), which dephosphorylates MEK1/2. Accordingly, p38 activation is followed by a reduction in ERK phosphorylation, and the opposite response is observed after inhibition of p38 activity [28–31]. ERK has antagonistic effects on JNK activation *in vitro* and *in vivo* [32,33]. In human alveolar macrophages, the interaction between them is mediated by MKP-7, a JNK phosphatase also known as dual specific phosphatase 16 (DUSP16). DUSP 16 is reduced after ERK inhibition, which is followed by increased JNK phosphorylation [32]. In Sprague-Dawley rats, induced-ischemia increases ERK and decreases JNK phosphorylation in the CA1 area of the hippocampus, but when animals are treated with a MEK inhibitor, JNK activity is augmented [33]. The inverse relationship is also possible, since JNK negatively regulates both ERK and p38 activation, as reported for mouse cardiomyocytes [34].

PI3K and MAPKs interaction can occur at different steps. At initiation level, PI3K-induced PIP3 recruits scaffold proteins to the plasma membrane, including GAB, which induces Grb2-SOS relocation to the membrane and consequently increases Ras activation [35]. At intermediary cascade level, Akt is able to phosphorylate and inhibit the activity of ASK1 and MLK3 that belong to the MAPKKK upstream activation of JNK, and the final effect is the decrease of ASK1- and MLK3-mediated cell death [36,37]. Conversely, the inhibition of PI3K-Akt in cultured cerebellar granule cells (CGCs) also decreases ASK1 phosphorylation, but this event is followed by an increase of p38 activity, while JNK remains at control levels [38].

Smads are the signaling molecules activated in the transforming growth factor β (TGFβ) canonical pathway, but upon stimulation, this family of peptides can induce the activity of ERK1/2 in different cell types [39–41]. In non-transformed fibroblasts, TGFβ triggers PI3K signaling by inducing p21-activated kinase 2 (Pak2) to phosphorylate c-Raf, that ends in ERK1/2 activation [40]. ERK5 is also activated by TGFβ in proximal tubular epithelial cells [42] and hepatocytes [43]. In cultured human corneal endothelial cells, the cooperative interaction between TGFβ and p38 was shown to regulate cell migration [44]. Additionally, ERK1/2 can also suppress Smads signaling through phosphorylation of specific sites in the linker region [45,46]. Bone morphogenetic proteins (BMPs) are members of the TGFβ family that phosphorylate and activate Smad1, causing its accumulation in the nucleus and subsequent transcriptional activity [45], which is inhibited by ERK1/2 [47].

Wnt/β-catenin signaling participates in normal embryonic development and cellular functions in adult tissues, but its deregulation has been implicated in tumor progression. This pathway interacts with different members of MAPK family, such as ERK1/2, JNK and p38 that are able to phosphorylate the LDL-related protein 6 (LRP6), which is a co-receptor of Wnt, and so their activation promote Wnt/β-catenin signaling [48]. This is actually one of the RTKs mechanisms that trigger Wnt/β-catenin cascade, besides its direct activation by β-catenin. As an example, upon FGF2 stimulation, ERK1/2 induces LRP6 phosphorylation, resulting in increased Wnt/β-catenin function [49]. Although these experiments show a cooperative interaction between these two transduction pathways, ERK1/2 can also be negative to Wnt/β-catenin system. Accordingly, the inhibition of MEK in A375 human melanoma cells, which present BRAF mutation and constitutive activation of ERK1/2, leads to increased Wnt/β-catenin signaling and higher apoptotic indices [50]. Adding to ERK1/2, p38 and JNK also communicate with Wnt/β-catenin pathway. The binding of Wnt3a to receptor FZ1 in mouse teratocarcinoma F9 cells rapidly increases p38, which stimulates Wnt/β-catenin cascade by reducing its proteasome-mediated degradation [51]. The same conditions also activate JNK and it cooperates with Wnt/β-catenin signaling [52].

Finally, we should mention the complexity of networks operating in stem cells, as in human embryonic stem cells (hESC), the interactions among PI3K, Smad2/3, ERK1/2 and Wnt/β-catenin can determine the cell fate into self-renew or differentiation [53].

## 4. MAPK and Epithelial Cell Proliferation and Differentiation

Epithelial cell proliferation and differentiation comprehend dynamic processes that are part of kinetic events during growth, maturation and function of tissues and organs. MAPK family members are important for cell cycle control in epithelial cells [54–57] that immediately respond to the binding of ligands to RTKs present in the plasma or internal membranes after endocytosis [4,58–60]. Because of the plethora of effects triggered by MAPK network in cells, we will drive our attention to the rapidly renewing epithelia that cover the gastrointestinal tract and focus on the importance of this pathway to some aspects of proliferation and differentiation in diverse cell types.

### 4.1. MAPKs and Gastric Cells

The epithelium that lines the gastric mucosa in the corpus region of the stomach is composed of six different populations which are arranged to form tubular glands that open to the lumen [61]. Though detailed studies have been conducted in the past, only recently the origin of these epithelial cells could be more dissected, allowing a closer observation of stem cell niche [61–65]. The gastric gland grows during pre- and postnatal development [66], and the embryonic organ fate depends on the mesenchyme-induced inhibition of Wnt signaling [61,67,68], which determines the morphogenesis of the corpus region of the stomach [61]. Importantly, several effects described for cell proliferation and differentiation depend on the function of epidermal growth factor receptor (EGFR) [69–72] that responds both to EGF and TGFα [73–76] and signal through MAPKs [77].

MAPK-driven cell proliferation in the gastric epithelium leads to growth during development, renewal in adult tissues, healing of ulcers and hyperproliferative disorders such as Ménétrier disease and cancer [15,72,78–80]. The differential output depends on many factors that include the frequency and magnitude of MAPK signaling, which are important in the control of cell cycle progression [81]. A recent report showed that DUSP5, which is a controller of these ERK1/2 properties, is down-regulated in gastric cancers, allowing the cascade to stimulate proliferation continuously [82].

During different developmental stages, MAPKs and their partners may also be involved in the modulation of key molecules that control the cell cycle. The inhibition of EGFR reduces the phosphorylation of ERK1/2 and alters the concentration of p21waf1 and p27kip1 in the gastric mucosa of early-weaned rats [72]. In aged animals, increased gastric cell proliferation is concomitant with augmented expression of EGFR and TGFα [83], which stimulate MEKs and ERK1/2 phosphorylation and control DNA synthesis through the regulation of AP-1 [15].

**Figure 2:**
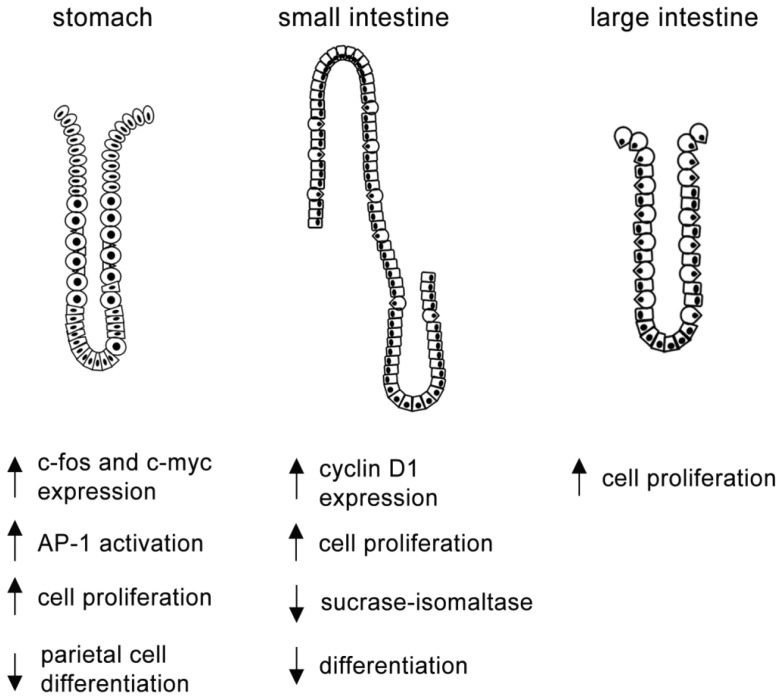
Effects of ERK1/2 activation in the gastrointestinal epithelium.

MAPKs are responsive to a broad range of proliferative stimuli. High transmural pressure loaded to gastric mucosal cells induces c-fos and c-myc expression, AP-1 activation and cell proliferation through phosphorylation of ERK1/2 (Figure 2), but not through JNK or p38 [84]. In addition, gastric cell proliferation induced by leptin, EGF and sonic hedgehog are impaired after inhibition of ERK1/2 signaling [85–87]. Besides ERK1/2, the MAPKs JNK and p38 also regulate gastric cell division. Accordingly, low intracellular chloride concentration induces p21 expression and inhibits the proliferation of human gastric adenocarcinoma MKN28 cells through activation of p38 and JNK, but not through ERK1/2 pathway [88].

As mentioned above, MAPK also drives cell proliferation under pathological conditions. *Helicobacter pylori* infection has been associated with high risks of chronic gastritis, peptic ulcer and tumorigenesis [89]. In gastric epithelial cells, the phosphorylation of ERK1/2, JNK and p38 is rapidly induced by *H. pylori* infection and these MAPKs promote differential effects on cell cycle progression and proliferation [90]. Ménétrier’s disease is another important pathology in the stomach, and it is characterized by hyperplasia of surface mucous cell, reduction of parietal cells population and increased expression of TGFα [79]. The therapy with monoclonal antibodies against EGFR ameliorates the symptoms and leads to a decrease in cell proliferation, which is triggered by low levels of ERK1/2 phosphorylation [91,92]. Additionally, Akt activation is augmented and the number of parietal cells is increased [91], which is consistent with the role of Akt and ERK signaling pathways in parietal cell functional maturation described below.

The differentiation of gastric cell populations involves the expression of morphogenetic proteins [65,93–96] that combined to the action of growth factors, hormones and nutritional elements start diverse signaling networks [71,97–99]. MAPK systems are essential in this complex mechanism. In parietal cells, the conjunction of plasma membrane, cytoskeleton and scaffold proteins is essential to maintain the activity of H^+^/K^+^ ATPase pump and the rearrangement of these elements depends on c-AMP mediated PKA signaling [100–102]. However, the differentiation of these cells seems to be governed by the activation of Akt pathway [103] that drives the inhibition of MAPK signaling in response to EGF [99] (Figure 2).

### 4.2. MAPKs and Intestinal Cells

The epithelium that covers the intestine is organized into crypt-villus structures in the small bowel or exclusively into crypts in the ceacum and colon segments. The single-layered intestinal epithelium is the most rapid cell renewal system in the organism, and under normal conditions it presents a turnover of 5 days. Due to the relevance of such property, numerous studies have been conducted recently to report and discuss the characteristics and functions of stem cell niche in the intestinal crypt [104–108]. From these stem cells, five different populations arise after proliferation and commitment to the differentiation of lineages. Organ fate is determined during embryonic development and depends directly on Wnt/β-catenin signaling [109–111].

Although the current focus in the literature is more onto the interactions of MAPK with Wnt/β-catenin in tumorigenesis [112,113], MAPK family members are regularly activated in the rapid dividing intestinal cells. The output of signaling, however, depends on the duration of stimuli, in way that high levels of p42/44 MAPK activity trigger cell proliferation, whereas low and sustained activation leads to differentiation [114]. Glucagon-like peptide 2 (GLP-2), peanut lectin and arginine vasopressin are among the stimuli that induce ERK1/2 signal to mediate intestinal epithelial proliferation [115–117]. In addition, apo-lactoferrin (iron-binding glycoprotein) increases cyclin D1 expression after ERK1/2 phosphorylation and promotes cell cycle progression in the mouse crypt [118] (Figure 2). The deletion of p38α in the colonic mucosa enhances the tumorigenesis induced by azoxymethane and dextran sodium sulfate in mice, suggesting a role for this MAPK as a cell cycle inhibitor under physiological conditions [119].

Among the RTKs binding molecules, EGF is one of the most important factors involved in intestinal growth, and responses are driven by MAPKs. EGF induces cell proliferation and inhibits apoptosis in cultured intestinal progenitor cells from mouse and human through activation of PI3K and ERK1/2 signaling pathways [120]. Moreover, when EGF stimulus is removed, fetal and adult intestinal progenitor cells differentiate [120]. Similarly to the gastric mucosa, aging leads to increased cell proliferation and activation of EGFR in the colonic mucosa [121,122]. In this condition, partners of

MAPK family, such as PI3K and Akt are highly active and cell proliferation in the colon overtakes cell death, increasing survival [123]. MAPKs are also involved in the differentiation of intestinal cell populations, and the synthesis of brush border enzymes is an important feature to trace the development and maturation of the organ (Figure 2). Accordingly, from MAPK family, p38 is activated in Caco2-15 cells and induce the transcription of sucrase-isomaltase enzyme through the regulation of CDX2/3 [124], which is essential in intestinal differentiation [125]. ERK1/2, however, has an opposite role in this process, and its inactivation is necessary for proper CDX2/3 transcriptional activity [126].

## 5. Nutritional Influence on Cell Signaling

Since birth, the epithelium that lines the mammal gastrointestinal tract interacts with luminal molecules present in colostrum and milk, and after weaning, in solid food. Besides its immunological relevance, milk contains growth factors and hormones [127] that are important to the growth control of the mucosa [71,72,128–131]. The effects triggered by feeding conditions, patterns and nutrient source affect directly epithelial cell proliferation and differentiation, which, as mentioned above, are greatly regulated by MAPKs.

During suckling period, EGF and TGFβ are examples of peptides that are continuously offered to neonates [127,132–134]. Although their physiological relevance in milk has been debated, both EGF and TGFβ are able to activate the respective signaling cascades through MAPKs and Smads in different organs [71,72,129,130,133]. In addition, disturbances of suckling change the stimuli and modify the output of MAPKs pathway, in a way that during early weaning, ERK1/2 and Src phosphorylation are amplified in gastric cells, and such condition results in increased cell proliferation and differentiation [71,72], and may contribute to the functional induction of ornithine decarboxylase in the gastric epithelium [135].

The nutritional status is another important element to regulate signaling in cells. Fasting (short-term food restriction), starvation (long-term food restriction) and feeding induce opposite cellular responses in terms of metabolism and growth, and MAPKs are their partners are directly involved in these regulatory mechanisms. In the hypothalamus, fasting stimulates ERK1/2 and p38 [136], whereas re-feeding re-establishes homeostatic conditions [137]. The specific control of these neuronal effects is still not completely understood, but the stimuli through corticosterone, ghrelin and other peptides are being explored. Adding to that, the gastrointestinal epithelium is also an important area in the regulation of nutrient uptake and it is affected by fasting/refeeding conditions. In the developing stomach, fasting stimulates cell proliferation [138,139] and though corticosterone has been described as part of such effect [140], MAPK action has not been identified yet. In the proximal duodenum, feeding regulates the expression of menin (a 67 kDa protein involved in metabolic control and tumor progression), that in turn inhibits PI3K-Akt signaling, resulting in decreased glucagon-like peptide 1 (GLP-1) secretion and reduced glucose-stimulated insulin release [141]. MAPKs are combined to these pathways in the control of glucose levels, especially through JNK that is extremely decreased or even lost in insulin-resistance [142,143].

Finally, the supplementation of diet with molecules that may have a protective effect in terms of tumorigenesis is being more deeply studied and focused on cellular signaling responses. For the rapid cycling intestinal epithelium, curcumin was compared to RTKs inhibitors and was shown to potentiate the decrease of growth and transformation of colon cancer cells [144], and the effect seems to occur through inhibition of MEK phosphorylation, which blocks ERK activity [145]. In lung injury induced by cigarette smoke, apple polyphenol and resveratrol were able to reduce the phosphorylation of p38 to levels equivalent to dexamethasone treatment [146].

Therefore, the therapeutical action of feeding and nutrients (currently identified as nutriceutical therapies) directly involve the triggering of MAPKs pathways, and altogether this will be a fruitful research field in the near future.

## 6. Conclusion

In this review, we reported how the major MAPKs and their communications with each other and with additional pathways create signaling networks that are important for cell fate determination. Moreover, we described how growth factors activate MAPKs and their partners to regulate the proliferation and differentiation in the rapidly renewing epithelia that line the gastrointestinal tract, and finally how nutrients affect MAPK transduction cascades. Therefore, we herein showed that although we tend to think about signaling mostly at molecular and cellular levels, when we consider the major effects in the gastrointestinal tract and impacts of these fast events, we find a broad field that has to integrate physiology, morphology, nutrition and cell biology.
